# Social isolation and loneliness: an integration framework for medical education

**DOI:** 10.3389/fmed.2026.1813649

**Published:** 2026-06-02

**Authors:** Jerlinda G. C. Ross, Veronica Escamilla, Carrie Elzie

**Affiliations:** 1 Methodist University Cape Fear Valley Health School of Medicine, Fayetteville, NC, United States; 2 Alice L. Walton School of Medicine, Bentonville, AR, United States

**Keywords:** curriculum development, health equity, loneliness, medical education, social determinants of health, social isolation

## Abstract

Loneliness and social isolation are increasingly recognized as critical public health issues, linked to adverse outcomes such as cardiovascular disease, cognitive decline, depression, and increased healthcare utilization. Despite their significance, these factors are underrepresented in medical education curricula. This Perspective advocates for the integration of loneliness and social isolation into undergraduate medical education (UME), emphasizing their roles as social determinants of health and their disproportionate impact on marginalized populations. We propose a multifaceted curricular framework encompassing foundational knowledge, clinical skills, innovative interventions, interprofessional education, and reflective practice. We provide strategies for addressing social isolation and social connection in medical education curricula using literature-based tools and experiential learning activities. By equipping future physicians with the competencies to address social disconnection, medical education can play a pivotal role in advancing health equity and improving patient outcomes.

## 1 Introduction

Loneliness and social isolation are increasingly recognized as critical public health challenges, with mounting evidence linking them to a broad range of adverse health outcomes, including cardiovascular disease, neurocognitive decline, depression, anxiety, premature mortality, and increased healthcare utilization (1). In 2023, the U.S. Surgeon General identified social disconnection as a national health priority, citing its widespread prevalence and substantial impact on individual and population health (1). Despite these urgent concerns and their relevance across clinical contexts, loneliness and social isolation remain underemphasized in undergraduate and graduate medical education (2).

As the practice of medicine continues to evolve toward population health, health equity, and biopsychosocial models of care (3, 4), the omission of these issues from formal curricula presents a critical gap. Preparing future physicians to understand and address the root causes of disease must extend beyond traditional biomedical risk factors to include the social conditions that fundamentally shape health (1, 3, 5). To this end, the deliberate integration of loneliness and social isolation into medical education is both timely and essential, not only to enhance clinical competency but also to support the development of a socially accountable healthcare workforce (5).

## 2 Framing loneliness and social isolation as social determinants of health in medical education

Within the broader framework of social determinants of health (SDOH), loneliness and social isolation merit explicit recognition (1). SDOH refer to the non-medical factors, such as economic stability, neighborhood environment, education, and social context. These non-medical factors influence health outcomes and drive inequities (1, 5). Loneliness, defined as the subjective feeling of insufficient or inadequate social connection, and social isolation, the objective absence of social relationships, are distinct but overlapping constructs (1, 5, 6). Both exert measurable effects on physical and mental health, comparable in magnitude to well-established risk factors such as smoking, obesity, and hypertension (6).

Framing loneliness and social isolation as SDOH elevates their importance in clinical reasoning, public health strategy, and medical education (5). This conceptualization aligns them with other priority areas such as food insecurity, housing instability, and structural racism, which positions them as critical domains of inquiry and intervention (7, 8). Incorporating validated screening tools into the curriculum can further introduce students to capturing loneliness and social isolation as part of clinical care. Tools such as the UCLA loneliness scale (9) and the PROMIS Bank v2.0-Social Isolation Short Form (10) have been used in clinical settings, including among patients with chronic obstructive pulmonary disease (11), heart failure (12), and older adults with cancer (13).

At the same time, it is important to acknowledge that definitions of loneliness and social isolation, and the ways they are expressed or recognized, may differ across cultural contexts (5). For medical students, this means learning to approach these constructs with cultural awareness, and to communicate with sensitivity (3, 4) Training that encourages learners to recognize how their own assumptions or biases can further strengthen their ability to assess social disconnection in ways that are equitable and responsive to diverse lived experiences (3, 14). Embedding culturally grounded case examples and opportunities to explore how loneliness and social isolation are understood within different communities can strengthen their ability to provide equitable, person-centered care (5, 15).

Moreover, because these experiences disproportionately affect older adults, individuals with chronic illness, people with disabilities, racial and ethnic minorities, and LGBTQ+ populations, addressing them is integral to any effort to reduce health disparities and promote health equity (5, 16). As such, medical education must equip learners to recognize, screen for, and respond to social disconnection as a routine and essential aspect of patient care (2, 3).

## 3 Curricular integration strategies

**FIGURE 1:**
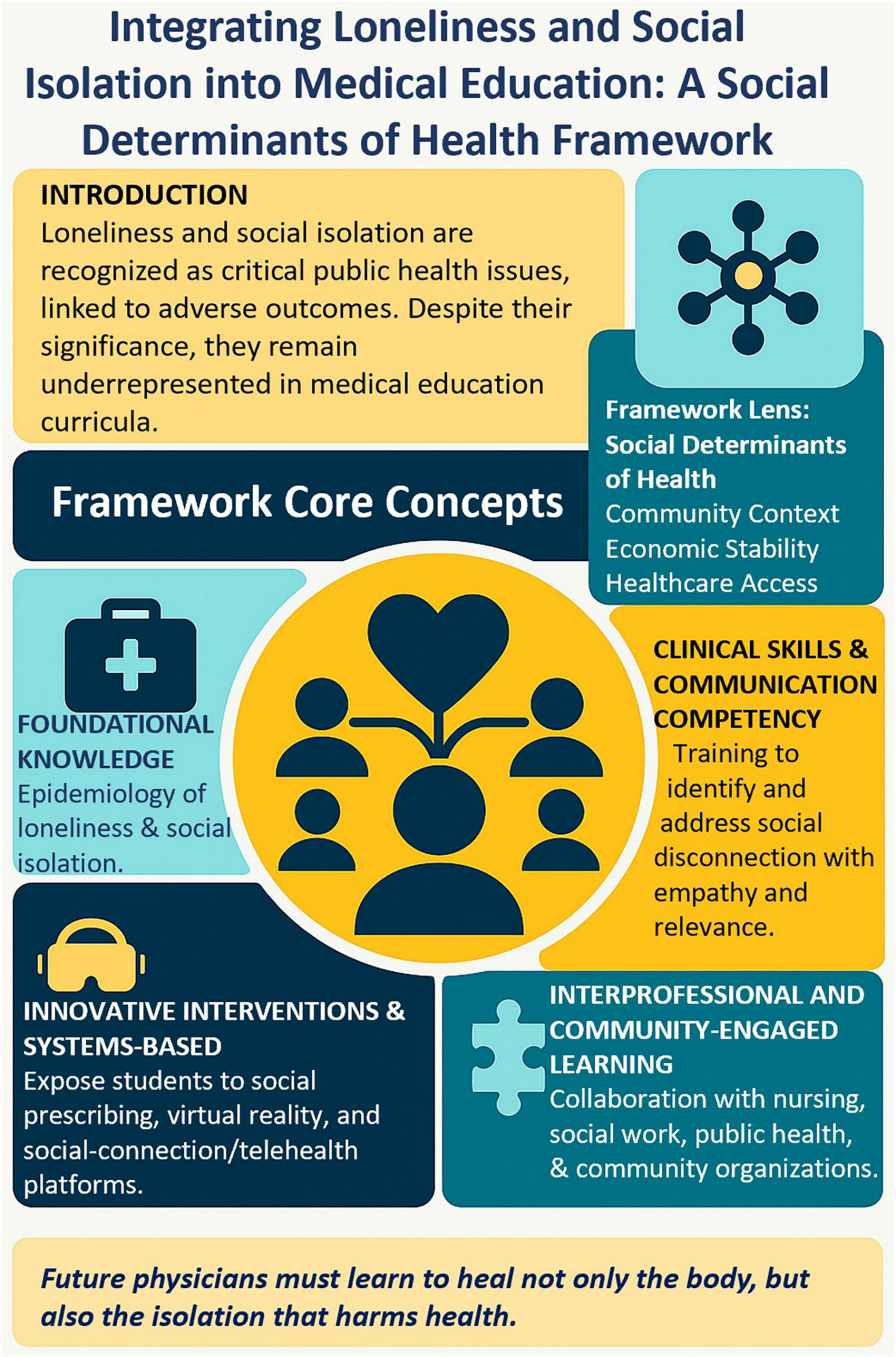
Curricular framework for integrating loneliness and social isolation into medical education. This infographic outlines a proposed educational framework to integrate the topics of loneliness and social isolation within medical education. It highlights these factors as critical social determinants of health and presents strategies across foundational knowledge, clinical skills, innovative interventions, and interprofessional learning to advance health equity and improve patient outcomes.

Recognizing loneliness and social isolation as critical social determinants of health establishes a compelling rationale for their deliberate inclusion in undergraduate and graduate medical education. However, acknowledgment alone is insufficient. Meaningful integration requires strategic curricular design that translates awareness into competence (2). To prepare future physicians to respond effectively to these pervasive issues, medical educators must adopt a comprehensive, multidimensional approach that spans the continuum of learning, from foundational knowledge and clinical skill development to interprofessional collaboration and reflective practice. Figure 1 outlines key strategies for integrating loneliness and social isolation into the medical curriculum. The key strategies include incorporating the impacts of loneliness and social isolation on health into foundational medical education, clinical skills and communication, introducing students to innovative interventions such as social prescribing to improve social connection, and collaboration with other fields, including nursing and community organizations for interprofessional and community-engaged learning. These strategies are presented within the lens of loneliness and social isolation as part of SDOH (Figure 1) and are further developed in the following sections, where we highlight approaches that are clinically relevant, equity-informed, and aligned with the broader goals of holistic, person-centered care.

### 3.1 Foundational knowledge and biomedical context

An effective integration of loneliness and social isolation into medical education must begin with a strong foundation in their biomedical and public health dimensions (5). Didactic content should introduce learners to the epidemiology of social disconnection, highlighting prevalence trends across age, gender, race, and socioeconomic strata, as well as its disproportionate burden on marginalized populations (5). These topics may be embedded within existing courses in public health, population medicine, behavioral science, or health systems science to emphasize their clinical relevance.

In addition, students should be exposed to the pathophysiological mechanisms that link social disconnection to adverse health outcomes (16). This includes instruction on the neuroendocrine, immunologic, and cardiovascular correlates of chronic loneliness, such as dysregulated hypothalamic-pituitary-adrenal (HPA) axis activity and increased proinflammatory cytokines (16). These mechanisms help explain associations with conditions such as hypertension, coronary artery disease, depression, cognitive decline, and increased all-cause mortality (16). Framing loneliness and isolation within a biopsychosocial model deepens students’ understanding of how upstream social experiences can produce downstream biological consequences and reinforces the importance of addressing these issues as part of routine clinical care (1, 2, 5).

Table 1 presents existing studies that support the incorporation of loneliness and social isolation into foundational UME content. For example, using a large survey design, Cudjoe et al. (17) identified a higher prevalence of social isolation among marginalized groups, including racial minorities, senior adults, and individuals with lower socioeconomic status. Paul et al. (18) demonstrated the relationship between chronic loneliness and increased risk of cardiovascular events, and Jafferany et al. (19) identified a feedback loop between skin disease and social isolation that contributes to a lower quality of life. Together, these studies (Table 1) can inform the development of didactic content that highlights the impact of loneliness and social isolation on health outcomes.

**TABLE 1:** Strategies to teach, assess, and engage learners in addressing social isolation and social connection in medical education.

Foundational sciences		References
Epidemiology	Include the epidemiology of social disconnection, presenting data on prevalence including how various groups of individuals (racial minorities, senior adults, lower socioeconomic status) often face a disproportionate burden due to systemic factors.	Cudjoe et al. (17)
Behavioral science/neuroscience	Through a case study of a patient experiencing chronic loneliness analyze how their social experiences (e.g., recent loss, lack of community) interact with psychological factors (e.g., depression, anxiety) and biological responses to impact their overall health, integrating discussions on the neuroendocrine correlates of chronic loneliness such as dysregulated hypothalamic-pituitary-adrenal (HPA) axis activity.	Zilioli et al. (29)
Cardiovascular	Discuss how chronic loneliness can contribute to increased sympathetic nervous system activity, elevated blood pressure, and increased risk of cardiovascular events.	Paul et al. (18)
Immunology	Correlate proinflammatory cytokines (e.g., IL-6, TNF-alpha) and altered immune cell function observed in chronically lonely individuals. Explain how this chronic low-grade inflammation can contribute to various diseases.	Pourriyahi et al. (30)
Health systems	Discuss how social isolation can create barriers to healthcare access, medication adherence, and follow-up care. Explore the role of telehealth or community health workers in reaching isolated individuals.	Hernández-López et al. (2); National Academies of Sciences, Engineering, and Medicine (5)
Dermatology	Social isolation is often a product of chronic skin disease and leads to impairment in the overall psychological wellbeing of patients making them more likely to experience depressive symptoms, social isolation, loneliness, and lower quality of life. The neuro-immune-cutaneous-endocrine (NICE) model explains how various neuromediators, hormones, cytokines, and feedback loops interact to regulate skin functions.	Jafferany et al. (19)

This table provides examples of how to incorporate loneliness and social isolation into undergraduate medical education (UME) curricula. This includes studies that provide foundational knowledge on the impact of loneliness and social isolation on health outcomes and access to care.

### 3.2 Clinical skills and communication competency

Beyond foundational knowledge, clinical education must prepare learners to recognize and respond to loneliness and social isolation as part of routine patient care (15). This requires explicit training in both the identification of social disconnection and the communication strategies needed to address it with empathy and clinical relevance (2, 15). Incorporating validated screening instruments such as the UCLA Loneliness Scale, the Lubben Social Network Scale, or single-item loneliness screeners into clinical skills curricula can equip students with structured tools for assessment (1, 5, 9, 20). These instruments can be introduced during physical diagnosis courses or longitudinal clinical skills sessions, reinforcing the idea that social context is as assessable and actionable as physical symptoms (2).

Equally important is developing the communication competence necessary to engage patients in meaningful conversations about social connection, emotional wellbeing, and support systems (5). Instruction in approaches such as motivational interviewing, active listening, and narrative medicine can enhance students’ confidence and fluency in addressing these sensitive topics (3, 4). Standardized patient encounters, role-playing, and reflective exercises provide opportunities for skill development and formative feedback in a safe learning environment (2). For example, a high-fidelity clinical simulation designed to address loneliness and social isolation in older adults via telehealth improved knowledge and attitudes towards older people among nursing students (2). Training modules included communication techniques and reminiscence therapy to prepare students for telehealth simulations (2). Another study demonstrated the value of communication training by connecting medical and nursing students to stroke survivors experiencing loneliness and social isolation through a social phone program (21). Students completed Good Clinical Practice and empathy-based communication training before making phone calls and wrote journal entries following phone calls. Analysis of student journal entries highlighted the importance of learning opportunities to support patients, raise awareness of post-stroke challenges including social isolation and loneliness, and provide holistic, compassionate care (21). Embedding these competencies early and longitudinally within clinical education helps ensure that future physicians are not only aware of the clinical significance of social disconnection but are also equipped to address it with professionalism, compassion, and cultural humility (5).

### 3.3 Innovative interventions and systems-based practice

Equipping future physicians to address loneliness and social isolation effectively requires a shift beyond conventional clinical interventions toward innovative, system-oriented strategies (1). Medical education should expose learners to emerging models of care designed to mitigate social disconnection, such as social prescribing, peer support networks, and technology-facilitated solutions (1). Social prescribing, already implemented in several international health systems, involves referring patients to non-clinical community resources, such as volunteer programs, group activities, or cultural engagement, to enhance social well-being and connectedness (22). Its integration into clinical workflows reflects a growing recognition of community-based assets as extensions of healthcare (5).

Similarly, students should become familiar with digital and technology-enabled approaches, including virtual reality applications, teleconnection platforms, and digital companionship tools that support social engagement among populations at high risk for isolation, such as older adults and individuals with limited mobility (5). Exploring these innovations within the curriculum not only broadens students’ awareness of available interventions but also fosters systems-based thinking, a core competency in contemporary medical education (3, 4).

By understanding how healthcare interfaces with social infrastructure, learners are better prepared to design, advocate for, and implement holistic, person-centered strategies that address the social dimensions of health across diverse care settings (5).

Integrating interventions and systems-based practices that address loneliness and social isolation into medical education curricula can be done through a series of focused modules. The first module should introduce students to existing evidence by reviewing and critically assessing studies that address loneliness and social isolation in clinical settings. Examples include an evaluation of clinical screening paired with an electronic health record-triggered resource referral (23), and an assessment of a simulation-based training designed to teach students to communicate effectively with patients experiencing loneliness and social isolation (2). A second module could focus on applied training to strengthen communication skills and practice implementing an intervention. This may involve participation in simulation-based exercises or engagement in phone-based volunteer programs that connect students to individuals experiencing loneliness and social isolation. Volunteering with existing programs and identifying community resources exposes students to the importance of community engagement and highlights its role in sustainable patient-centered interventions.

### 3.4 Interprofessional and community-engaged learning

Effectively addressing loneliness and social isolation requires coordinated, team-based solutions that extend beyond the scope of individual clinicians (5, 15). As such, interprofessional education (IPE) is an essential pillar of curricular integration (24). Structured learning experiences that bring together students from nursing, social work, public health, psychology, and other allied health professions foster a shared understanding of the complementary roles each discipline plays in supporting patients’ social and emotional wellbeing (24). These collaborations cultivate essential competencies in teamwork, communication, and role clarity, building skills that are foundational to comprehensive, person-centered care (3, 4).

In parallel, community-engaged learning opportunities offer medical students direct, meaningful interactions with populations most vulnerable to social disconnection (5). Longitudinal service-learning partnerships with long-term care facilities, home-based care programs, senior centers, or community-based organizations enable students to witness firsthand the lived experiences of isolation, while also contributing to interventions that promote social connection (5, 15). Programs that connect medical students with older adults experiencing loneliness and social isolation have shown educational value (25, 26). Incorporating participation in these outreach programs into the curriculum underscores the importance of community engagement in addressing patient needs holistically. These experiences deepen empathy, reinforce cultural humility, and help shape a professional identity grounded in social accountability and equity (3, 4). By engaging learners in both interprofessional collaboration and community service, medical education can cultivate a generation of physicians equipped not only to identify social isolation as a health risk but to respond in ways that are inclusive, collaborative, and sustainable (24).

### 3.5 Evaluation, reflection, and professional formation

To ensure that the integration of loneliness and social isolation into medical education leads to lasting attitudinal and behavioral change, curricula must include structured opportunities for reflection, assessment, and professional identity formation (3, 14). Because loneliness is also prevalent among health care workers (27) and medical students (28), students should be encouraged to reflect on their own experiences and be informed of available formal support resources, such as peer mentoring or counseling services.

Incorporating experiences in which students engage directly with patients experiencing loneliness provides opportunities for learning, reflection, and evaluation. This can include keeping a journal of their interactions to reflect on the experience and what they learned (21). Embedding reflective writing assignments, small group discussions, and facilitated debriefings allows students to process their experiences engaging with socially isolated patients and to examine their own assumptions about social connection and vulnerability (3, 14). This form of reflection not only enhances emotional intelligence and empathy but also reinforces the importance of addressing social determinants of health as a core professional responsibility (5, 15).

In addition, formative assessments, including standardized patient encounters, clinical observations, and narrative evaluations, can be used to assess learners’ competencies in identifying and responding to social disconnection within patient care (2, 3). Pre- and post-surveys can be used to evaluate student competencies following training and participation in patient outreach and care, including knowledge of social isolation and perceived value of addressing it in patients (26), and the ability to recognize and reduce biases after working with older adult patients (25). These methods provide meaningful feedback and support iterative growth over time (2, 3). Longitudinal assessment strategies, such as portfolios or milestone-based evaluations, can further track learners’ development in integrating social context into their clinical reasoning and patient interactions (3, 4). Together, these reflective and evaluative practices promote the internalization of key concepts and foster the emergence of a professional identity that prioritizes relational care, equity, and human connection as essential components of high-quality healthcare (3, 4).

## 4 Advancing health equity

The curricular strategies outlined above are not only essential for cultivating clinical competence; they are also critical levers for advancing health equity (5, 8). Loneliness and social isolation are not experienced uniformly across populations; rather, they disproportionately impact historically marginalized groups, including older adults, individuals with disabilities, racial and ethnic minorities, LGBTQ+ populations, and those living in poverty or under-resourced communities (5). These disparities are compounded by structural inequities that limit access to social support, community engagement, and culturally responsive care (5, 8).

By equipping future physicians with the knowledge, skills, and perspectives needed to recognize and respond to social disconnection, medical education can actively contribute to the reduction of inequitable health outcomes (3, 4). Integrating these topics into curricula affirms the centrality of social determinants in shaping patient experiences and reinforces the physician’s responsibility to address upstream drivers of health, both in individual encounters and through systems-level advocacy (5). This educational focus aligns with national efforts to reorient healthcare toward equity, prevention, and person-centeredness, ensuring that tomorrow’s physicians are prepared not only to treat disease but to build a more just and connected health system (1).

## 5 Conclusion

Integrating loneliness and social isolation into undergraduate medical education is not merely a curricular enhancement; it is an essential evolution in preparing future physicians to address the full spectrum of factors that shape health and well-being. These social conditions are powerful, often overlooked drivers of morbidity, mortality, and healthcare utilization, particularly among marginalized and high-risk populations. A comprehensive, multidimensional approach encompassing foundational biomedical knowledge, clinical communication skills, exposure to systems-based interventions, interprofessional collaboration, and reflective practice can equip future physicians to respond meaningfully and effectively to these pervasive challenges.

As medical education continues to advance toward deeper integration of social determinants of health, health equity, and person-centered care, loneliness and social isolation must be recognized not as peripheral concerns but as core to medical training (5, 8). Deliberate curricular strategies that foreground these issues help shape physicians who are attuned not only to disease but to the lived experiences of their patients. In doing so, medical education can play a transformative role in reducing health disparities, improving patient outcomes, and strengthening the human connection at the heart of clinical care.

## Data Availability

The original contributions presented in this study are included in this article/supplementary material, further inquiries can be directed to the corresponding author.
